# Prostate cancer in renal transplant recipients

**DOI:** 10.1590/S1677-5538.IBJU.2016.0510

**Published:** 2017

**Authors:** Benjamin A. Sherer, Krishnan Warrior, Karl Godlewski, Martin Hertl, Oyedolamu Olaitan, Ajay Nehra, Leslie Allan Deane

**Affiliations:** 1 Department of Urology, Rush University Medical Center, Chicago, Illinois, United States; 2 Department of Surgery, Abdominal Transplant, Rush University Medical Center, Chicago, Illinois, United States

**Keywords:** Prostate cancer, familial [Supplementary Concept], Kidney Transplantation, Prostatectomy, Radiotherapy, Prostate-Specific Antigen

## Abstract

As patients with end-stage renal disease are receiving renal allografts at older ages, the number of male renal transplant recipients (RTRs) being diagnosed with prostate cancer (CaP) is increasing. Historically, the literature regarding the management of CaP in RTR's is limited to case reports and small case series. To date, there are no standardized guidelines for screening or management of CaP in these complex patients. To better understand the unique characteristics of CaP in the renal transplant population, we performed a literature review of PubMed, without date limitations, using a combination of search terms including prostate cancer, end stage renal disease, renal transplantation, prostate cancer screening, prostate specific antigen kinetics, immuno-suppression, prostatectomy, and radiation therapy. Of special note, teams facilitating the care of these complex patients must carefully and meticulously consider the altered anatomy for surgical and radiotherapeutic planning. Active surveillance, though gaining popularity in the general low risk prostate cancer population, needs further study in this group, as does the management of advance disease. This review provides a comprehensive and contemporary understanding of the incidence, screening measures, risk stratification, and treatment options for CaP in RTRs.

## INTRODUCTION

In 2013, nearly 30.000 patients underwent solid organ transplantation in the United States, of which 16.894 were renal allografts (1). It is widely acknowledged that patients are receiving grafts at older ages and are experiencing longer life expectancies with sustained renal function. Treating these patients for non-transplant related conditions, including prostate cancer (CaP), has become more frequent. In this review, we provide a comprehensive and contemporary assessment of CaP risk, screening, and treatment effectiveness in the renal transplant population.

## MATERIALS AND METHODS

We performed a comprehensive literature review of articles published from January 1, 1989 through May 1, 2014 using PubMed/Medline and the Cochrane Collection. We utilized a pre-determined search strategy including the terms prostate cancer, end stage renal disease, renal transplantation, prostate cancer screening, prostate specific antigen (PSA) kinetics, immunosuppression, prostatectomy, and radiation therapy. All studies included were performed in adult human beings (>18 years old), written in English, and had full text obtainable for review.

## RESULTS

### Incidence

Compared to age-matched controls in the general population, transplant recipients are at an increased risk for a variety of malignancies. Overall, the 5-year incidence of cancer in solid organ transplant recipients is 4.4%, although hazard ratios vary based on age and organ transplanted (2). Among RTRs, genitourinary malignancies are the third most common malignancy behind de novo skin malignancies and post transplant lymphoproliferative disorder (3, 4). Of the genitourinary malignancies, CaP is the most common (5).

It remains a point of controversy as to whether RTRs are truly at increased risk of developing CaP. Recently reported standardized incidence ratios for CaP in solid organ transplant recipients are variable, ranging from 0.88-1.70 (6–10) (Table-1). Data from the 1980's and 1990's suggested that transplant patients were not at increased risk for CaP (3, 11). However, many theorize that CaP has become more frequent in the RTR population due to increased allograft survival, increasing recipient age, and more rigorous screening. Variability in reported incidence may also be attributed to differences in study design, geography, screening practices, reporting criteria, sample size, and the immunosuppressive regimen used (3, 6, 11–18).

**Table 1:** Standardized Incidence Ratio (95% confidence Interval) of Malignancies in Renal Transplant Recipients (6 – 10).

	Collett 2010 (6)	Cheung 2012 (7)	Vajdic 2006 (8)	Piselli 2013 (9)	Tessari 2013 (10)
Prostate	1.1 (0.9-1.4)	0.88 (0.39-1.95)	0.95 (0.68-1.29)	1.7 (1.2-2.3)	1.3 (0.8-2.1)
Lip	65.6 (49.9-84.6)	–	47.08 (41.75-52.89)	9.4 (3.1-22.0)	–
Esophagus	1.8 (1.3-2.5)	1.12 (0.28-4.49)	3.82 (2.26-6.03)	1.2 (0.3-3.6)	–
Stomach	2.0 (1.4-2.6)	2.85 (1.62-5.02)	1.84 (1.07-2.94)	1.4 (0.8-3.3)	1.1 (0.5-2.4)
Colorectal	1.8 (1.6-2.1)	1.75 (1.22-2.52)	2.36 (1.87-2.92)	0.8 (0.5-1.2)	1.2 (0.7-1.9)
Pancreatic	1.5 (1.0-2.1)	1.56 (0.41-4.87)	1.21 (0.56-2.30)	0.9 (0.3-2.0)	0.4 (0.2-1.8)
Liver	2.4 (1.5-3.8)	2.53 (1.63-3.91)	3.19 (1.53-5.87)	0.4 (0.1-1.1)	1.2 (0.5-2.7)
Melanoma	2.6 (2.0-3.3)	9.09 (2.27-36.34)	2.53 (2.08-3.05)	1.8 (0.9-3.3)	1.0 (0.4-3.0)
Non-Melanoma Skin Cancer	16.6 (15.9-17.3)	7.38 (4.86-11.21)	–	–	29.3 (26.0-33.1)
Kaposi sarcoma	17.1 (8.9-30.0)	–	207.90 (113.66-348.82)	135 (106–169)	84.9 (56.9-126.7)
Renal	7.9 (6.7-9.3)	12.5 (8.51-18.36)	7.30 (5.67-9.22)	4.9 (3.4-6.8)	7.0 (5.0-9.8)
Bladder	2.4 (1.9-3.0)	8.22 (4.67-14.47)	3.33 (2.40-4.50)	1.1 (0.7-1.7)	1.4 (0.8-2.5
Cervical	2.3 (1.4-3.5)	7.19 (3.87-13.37)	2.49 (1.33-4.27)	–	8.9 (4.4-17.7)
Uterine	1.0 (0.6-1.7)	1.44 (0.47-4.47)	1.74 (0.92-2.97)	1.3 (0.5-2.9)	1.1 (0.3-3.3)
Breast	1.0 (0.8-1.2)	1.66 (1.0-2.75)	1.03 (0.78-1.34)	0.8 (0.5-1.2)	1.2 (0.8-1.8)
Hodgkin's Lymphoma	7.2 (5.3-10.2)	–	3.75 (1.51-7.73)	2.3 (0.5-6.8)	1.0 (0.1-7.1)
Non-Hodgkin's lymphoma	12.5 (11.2-13.8)	15.79 (11.9-20.95)	9.86 (8.37-11.54)	4.5 (3.2-6.1)	7.9 (6.0-10.5)

More recent data indicates that renal transplant recipients do indeed have a higher incidence of CaP. Current U.S. Medicare data reveals a 3-year CaP incidence of 1.74%, which is significantly higher than age-matched controls in the general population (13). Similarly, data from 22 transplant centers in France has revealed a similar two-fold increase incidence of CaP (1.74%) in RTRs. Multiple studies have also verified that CaP is diagnosed earlier in RTRs (~62.3 years) versus the general population (70 years) (15, 18, 19).

Race may also play a role in CaP risk among RTRs. Hall et al. recently used data from the Transplant Cancer Match study to compare CaP risk in Caucasian, African American, and Hispanic RTRs. Much like the general population, African American RTRs have an increased risk of CaP, with a 2.14 incidence ratio compared to the Caucasian population (2).

### CaP Screening in renal transplant recipients

Best recommendations for CaP screening remain a point of contention in both the general and renal transplant populations. To date, there are no standard CaP screening regimens or established guidelines regarding prostate specific antigen (PSA) testing or cut-offs in pre or post—transplant patients. The American Society of Transplantation and the European Expert Group on Renal Transplantation do encourage annual CaP screening with digital rectal exam (DRE) and PSA measurement in all male RTRs who are older than age 50 and have a life expectancy of at least 10 years (13, 20). However, the validity of these recommendations has not been evaluated in a randomized controlled manner.

In centers that mandate strict CaP screening with a PSA threshold of >4ng/mL prior to biopsy, positive biopsy rates have been reported to be significantly higher than the general population, prompting some to suggest that lower PSA thresholds may be necessary in RTRs (12, 21).

It is unclear whether CaP screening is of benefit in the pre-transplant population, as the median survival for ESRD patients on hemodialysis is only 5 years and CaP is unlikely to cause significant mortality within that time period (22). However, post-transplant life expectancies now often reach well beyond 15 years, making the diagnosis and treatment of CaP in RTRs a realistic life-extending measure.

Yet, screening for CaP in RTRs may still be less cost effective and result in less overall benefit when compared to the general population. Kiberd et al. established computer simulation models to estimate life lost due to prostate, breast, and colorectal cancer in RTRs. Compared to the general population, it is estimated that three times more RTRs over age 65 must be screened with annual DRE and PSA to save one life (number needed to screen of 96 vs. 306, respectively) (23).

### PSA interpretation

An understanding of PSA kinetics is important when interpreting PSA values in ESRD and recently transplanted patients. Free PSA (f-PSA) is eliminated by the kidneys. Therefore, f-PSA and % f-PSA are elevated in ESRD patients. Serum f-PSA levels are significantly higher in hemodialysis patients, but are not significantly elevated in patients undergoing continuous ambulatory peritoneal dialysis. In contrast, % f-PSA is persistently elevated (by about 40%) in ESRD patients on any form of dialysis (24). After renal transplantation, f-PSA decreases significantly (by up to 60% within 6 days), especially in patients with better graft function as decline in f-PSA and % f-PSA correlate with decreases in serum creatinine (25, 26).

In contrast, total PSA (t-PSA) levels are more likely to reflect levels of complexed PSA (bound to α-1-antichymotrypsin or α-2-macroglobulin) which is metabolized by the liver. After renal transplantation, t-PSA levels are relatively unchanged. Therefore, t-PSA is the most reliable CaP marker in both pre-transplant patients on dialysis and in the early post-transplant population (24–26, 28–30). A summary of PSA kinetics and implications in RTRs can be found in Table-2.

**Table 2:** PSA Kinetics in Renal Transplant Recipients (31–35).

Marker	Metabolism	Half-Life	Variation in ESRD Patients (compared to normal)	Expected Change after Renal Transplantation
**t-PSA**	Hepatic	1.9 - 3.4 days	No significant change	No significant change
**f-PSA**	Renal	69 - 110 minutes	Increased	Decreased (30% in 24 hours, up to 60% in 6 days)
**%f-PSA**	Renal	–	Increased	Decreased
**f/T-PSA ratio**	Renal/Hepatic	–	Increased	Normal

Screening RTRs with t-PSA does seem to have important diagnostic implications; when t-PSA level is between 4-10ng/mL, there is only a 17% chance of CaP. If t-PSA levels exceed 10ng/mL, the risk of CaP increases to ~50% (29).

Although t-PSA does not seem to be significantly affected by renal transplantation itself, interpretation of PSA in RTRs may need to be adjusted based on immunosuppression regimen. Retrospective analysis by Chamie and colleagues has revealed that, among patients with similar PSA levels prior to renal transplant, post-transplant PSA was significantly lower in patients taking sirolimus (0.9ng/mL) versus tacrolimus (1.9ng/mL). It remains unclear whether sirolimus increases PSA elimination, decreases PSA production, or precludes the use of PSA as a CaP screening tool (36).

### Use of Imaging in ESRD patients and RTRs

Regardless of immunosuppressive regimen, CaP screening in RTRs at most centers includes digital rectal exam and t-PSA. Subsequent transrectal ultrasound guided biopsy is performed if t-PSA is abnormally elevated (27) Although not specifically studied in RTRs, multiparametric magnetic resonance imaging (mp-MRI) is emerging as an accurate tool for identifying clinically relevant prostate tumors. Mp-MRI can be used to identify significant lesions prior to a target biopsy or as a cancer staging tool after a positive biopsy (37). MRI can also help rule out CaP metastases and aid in identifying the location of important structures (namely the transplant allograft and the transplant ureter) prior to CaP treatment (38).

Although a high prevalence of nephrogenic systemic fibrosis in ESRD patients exposed to gadolinium-based contrast agents has been reported (10%), nephrogenic systemic fibrosis is almost exclusively seen in patients with a glomerular filtration rate (GFR) less than 15mL/min/1.73m^2^. Risk is minimal among patients with milder degrees of renal insufficiency (39, 40). To further minimize risk, it is generally recommended to avoid MRI contrast agents in patients with a GFR <30mL/min/1.73m^2^.

### Antibiotic prophylaxis for transrectal ultrasound (TRUS) guided biopsy in RTRs

American Urological Association guidelines recommend antibiotic prophylaxis in all patients undergoing TRUS-guided prostate biopsy. Multiple randomized controlled trials have shown a decrease in infectious complications of prostate biopsy with single-dose antibiotic prophylaxis. The antimicrobials of choice include fluoroquinolones or 1st/2nd/3rd generation and cephalosporins. Acceptable alternatives include trimethoprim/sulfamethoxazole or an aminoglycoside. Aztreonam can be used instead of an aminoglycoside in patients with renal insufficiency (41).

Although no specific guidelines exist for antibiotic prophylaxis in the undoubtedly higher risk transplant recipient, similar recommendations can be applied to this population. In small prospective studies, TRUS-guided prostate biopsy has been shown to be well tolerated in patients receiving immunosuppression, with no increased risk of infection compared to the general population (42). Nonetheless, careful consideration to culture directed prophylaxis may be crucial to minimizing the risk of infectious complications in RTRs exposed to multiple hospitalizations, previous exposure to multiple antibiotics, and concomitant immunosuppression (43).

When choosing a prophylactic regimen, it is also important to consider that one fourth of RTRs will develop a urinary tract infection within one year of transplantation. A urinalysis and culture should be checked and any active urinary tract infection should be fully treated prior to proceeding with prostate biopsy. Post-transplant TMP-SMZ prophylaxis is associated with a lower risk of UTIs in this population, but frequent use of TMP-SMZ in RTRs can lead to resistant organisms. Thus, in patients who are already on post-transplant TMP-SMZ prophylaxis regimen, a different class of antibiotic should be used for TRUS-biopsy prophylaxis in RTRs (44). Because RTRs represent a population at high risk for harboring resistant organisms, they may also benefit from pre-biopsy rectal swabs for directed prophylaxis.

### CaP Risk Stratification

The average stage at diagnosis of CaP in RTRs is no different than in the general population and ~84% of RTRs with CaP are diagnosed with localized disease. However, after diagnosis, CaP seems to progress more rapidly in RTRs and disease specific survival for stage II, III, or IV disease is significantly shorter compared to the general population (19, 45).

Risk stratification in RTRs with CaP may be heavily influenced by immunosuppression regimens. Use of immunosuppression has been linked to a variety of malignancies, but the association of immunosuppression and CaP is less clearly delineated. There are no large-scale comprehensive studies with adequate follow-up to assess whether immunosuppression truly increases risk of CaP occurrence, recurrence, or progression. Nonetheless, CaP is diagnosed at earlier ages in RTRs which may be due to more diligent screening or an actual increased predilection for CaP. Men with HIV on a variety of immunosuppression regimens also develop CaP at increased incidence and at earlier ages compared to the general population, indicating that immunosuppression may indeed be implicated (46, 47).

It has been postulated that a healthy immune system is essential for the inhibition of CaP cell growth. When CaP cell lines are exposed to a healthy conditioned media containing normal T-lymphocytes, they demonstrate decreased growth. Some experts propose that a suppressed immune system may disrupt the “normal” milieu of T-lymphocytes and lymphokines that are typically responsible for prevention of CaP development and progression (48).

CaP cells appear to respond differently based on the type of immunosuppressive agent introduced. In vitro studies and animal models suggest that calcineurin inhibitors (CNIs) increase aggressiveness and progression of CaP. Cyclosporine has been shown to induce phenotypic changes that make various forms of adeno-carcinoma more aggressive. In murine models of prostate adenocarcinoma, cyclosporine increases the size and number of lymph node and pulmonary metastases. The mechanism may be tissue growth factor beta (TGFB) mediated, as anti—TGFB antibodies have been shown to prevent these alterations (49, 50).

Azathioprine (AZA) has been strongly linked to an increase in skin malignancies and may also have a stimulatory influence on CaP cells. In 2008, a retrospective study of 19 French transplant centers evaluated immunosuppression in 62 RTRs with CaP. Patients on a CNI+AZA were more likely (45%) to be diagnosed with high stage (III and IV) CaP compared to CNI alone (15%) and had higher rates of metastatic disease. Use of AZA was the only independent risk factor for advanced disease in this cohort. However, other studies have shown no increased risk of CaP in association with AZA or mycophenolate (15, 51, 52).

Other immunosuppressive agents may be protective against CaP. In vivo, mammalian target of rapamycin (mTOR) inhibitors (sirolimus/rapamycin) have an inhibitory effect on the proliferation of CaP cell lines. As a downstream kinase in the Phosphatidylinositol-3/AKT signaling pathway, mTOR promotes cell survival and normal cell replication, and is often dysregulated in aggressive CaP cell lines. Therefore, inhibition of mTOR kinase and S6 kinase by mTOR inhibitors may prevent CaP cell cycle progression (53). Treatment of CaP cell lines with mTOR inhibitors has also been shown to increase the radiosensitivity of these cells by ~20% (54).

In 2005, Kauffman and colleagues evaluated data from 33.249 RTRs in the Organ Procurement and Transplant Network database and compared the identified occurrence of de novo malignancy in RTRs receiving either a mTOR inhibitor (sirolimus/everolimus), a CNI (cyclosporine/tacrolimus), or both. The incidence of de novo cancer was significantly lower in the mTOR inhibitor group compared to the CNI group (0.60% vs. 1.61%; p-value <0.001). The authors concluded that the use of mTOR inhibitors alone or in combination with other immunosuppressive agents may reduce the incidence of CaP in RTRs. Combination therapy is often used to reduce the toxicity of individual agents (55).

In conclusion, current data suggests that cyclosporine, tacrolimus, and AZA are associated with a higher risk of malignancy, which may include CaP. In contrast, mTOR inhibitors are associated with a decreased incidence of malignancy, and should considered for use in higher risk patients (56). Reduction of immunosuppression is frequently instituted after CaP diagnosis, but to date there is no data showing any benefit with regards to prognosis. It is postulated by some that the intensity of therapy, rather than choice of agent, is related to increased CaP risk. Therefore, the lowest possible dose of immunosuppression while maintaining rejection-free graft survival is advised (11).

### Treatment

A variety of treatment options exist for CaP in the general population, and most have been applied to RTRs with CaP. There are a number of important considerations when deciding on treatment approach, mainly because of the proximity of the allograft and transplant ureter to the surgical or treatment field. Of utmost importance is to avoid any compromise to the allograft, as survival at 5 years is only 60% if renal allograft function is significantly impaired (57).

Most surgical approaches for CaP have been described in RTRs, including open radical retropubic prostatectomy (RRP), perineal radical prostatectomy (PRP), laparoscopic radical prostatectomy (LRP), robotic assisted laparoscopic radical prostatectomy (RARP), and extraperitoneal robotic assisted laparoscopic prostatectomy (ERARP). A summary of surgical series reporting prostatectomy approaches and outcomes in RTRs is shown in Table-3.

**Table 3:** Radical prostatectomy in the Renal Transplant Recipient population (15, 17, 60–71).

**Radical Retropubic Prostatectomy (RRP)**									
Study	Surgery	Patients	Age	Pre-operative PSA (ng/mL)	Operative time (minutes)	Estimated Blood Loss(mL)	Length of Stay (days)	Graft injury/ impairment	Complications
Kinahan 1991 (17)	RRP	3	60	–	133	1466	10	none	2a
Kleinclauss 2008 (15)	RRP	20	60.4	7.1	163	516	11.9	1	4b
Thompson 2008 (60)	RRP	17	59	4.8	161	500	3	none	6c
Antonopoulous 2008 (70)	RRP	8	59.6	4.5	183	656	5	none	2 d
Hoda 2010 (57)	RRP	16	61.8	4.7	108.3	211.1	10.1	none	2 e
Total	RRP	64	60.26	5.49	149.89	497.56	9.99	1	16

**Perineal Radical Prostatectomy (PRP)**									
Study	Surgery	Patients	Age	Pre-operative PSA (ng/mL)	Operative time (minutes)	Estimated Blood Loss(mL)	Length of Stay (days)	Graft injury/ impairment	Complications
Yiou 1999 (61)	PRP	2	62.5	15	–	–	–	none	none
Hafron 2005 (62)	PRP	7	62.3	8.0	92.7	492.9	2.6	none	1 f
Total	PRP	9	62.34	9.56	92.7	492.9	2.6	none	1

a One patient with severe UTI; one patient with mild stress incontinence.

b One transplant ureteral stricture with associated allograft failure; three patients developed urosepsis.; one patient developed medium-volume lymphocele requiring percutaneous drainage.

c One patient with post-operative hemorrhage; one wound infection; one post-operative myocardial infarction; two patients had the late complication of incontinence at 1 year.

d Two patients with perioperative blood loss requiring transfusion of 2 and 6 units of packed RBCs respectively.

e One patient with prolonged hematuria requiring transfusion; one vesico-urethral anastomotic leak requiring prolonged Foley catheterization.

f One patient had prolonged gross hematuria requiring continuous bladder irrigation and transfusion of 1 unit of packed RBCs.

Regardless of approach, prostatectomy in transplant patients poses a number of unique challenges. Previous transplant surgery leads to distortion of normal anatomy and the renal allograft limits exposure within the pelvis. RTRs with a history of peritoneal dialysis are likely to have thickening of the peritoneum and obliteration of normal tissue planes. Performance of a bilateral lymph node dissection in RTRs may be difficult, dangerous, or nearly impossible as most allografts are oriented along the iliac vessels. In many instances, it may only be possible to perform a contralateral lymphadenectomy. Doing so is not without future risk; extensive lymphadenectomy may render the contralateral iliac vessels unusable for future allograft implantation should the current graft fail at a later date.

Despite these challenges, the literature supports the safety and efficacy of prostatectomy in the post-transplant population. Disease specific and overall mortality after aggressive surgical therapy in transplant patients is comparable to that in the non-transplant population.

### Active Surveillance

There is no data on active surveillance for prostate cancer in the renal transplant population. Conceptually, this may become an evolving arena in which the application of advanced biomarker evaluations and tissue genomics plays an increasing role in predicting who is a candidate for such an approach. In the future, multi-parametric magnetic resonance imaging (mpMRI) may shed some light on the presence or absence of prostate lesions of significance, provided the glomerular filtration rate permits the administration of gadolinium, which may help to guide decisions for intervention.

### Radical Retropubic Prostatectomy (RRP)

RRP, the traditional standard approach for localized CaP in the general population, was first reported in a RTR by Manson in 1989. Inherent dangers of RRP in RTRs became evident, including the insertion of deep retractors that can damage the allograft and ureter (58). Since that time, the safety and efficacy of RRP in RTRs is well supported.

Regardless of surgeon experience, visualization during RRP in RTRs is likely to be limited due to the pelvic renal allograft. French surgeons have noted operative dissection during RRP to be “difficult” in 35% of RTRs versus 20% in control patients. Specifically, dissection to achieve bladder descent is challenging due to a tethering effect from the transplant ureteroneocystostomy (59).

Most urologists avoid lymph node dissection ipsilateral to the transplant kidney to avoid the risk of allograft injury. However, bilateral lymph node dissection can be completed if deemed necessary. Kinahan and colleagues reported bilateral ileo-obturator lymph node dissection by using modified placement of Wilkinson retractors to gain exposure while preventing pressure damage to the transplant kidney (17). Placement of a transplant ureteral stent prior to prostatectomy may help with transplant ureteral identification if bilateral lymphadenectomy is to be performed.

In the early postoperative period, it is felt that immunosuppression does lead to delayed wound healing and may contribute to an increased risk of perioperative infection (60). French transplant centers have reported a significant increase in systemic (non-wound) perioperative infections (15% vs. 2.5% in nontransplant patients) (59). However, to date, no series of RRP in RTRs have shown a significant increase in wound infection.

Although RRP has been performed safely in RTRs with relatively efficient operative times, blood loss and hospital stays are significantly increased compared to laparoscopic and robotic assisted approaches (Table-3).

### Perineal Radical Prostatectomy (PRP)

Although technically challenging, PRP offers a few potential advantages over RRP or laparoscopic approaches. The main benefit is minimal dissection near the renal allograft and transplant ureter. With the perineal approach, bladder descent is not impaired and vesicourethral anastomosis can be performed without tension. PRP also preserves access to the iliac fossa should the need for a second transplant arise in the future. Therefore, PRP should be considered in RTRs with higher risk of impending graft failure. Small series report operative times comparable to RRP, while blood loss and length of stay tend to be lower (61, 62). PRP is limited to patients with low grade disease that does not necessitate a lymph node dissection. Another potential downside of this approach is the resulting perineal wound that may be at increased risk of infection or breakdown in immunosuppressed patients. Although ideologically the “safest” technique from the standpoint of allograft protection, widespread feasibility is impractical as most urologic surgeons are more familiar with RRP or robotic assisted approaches.

### Minimally Invasive Radical Prostatectomy: Laparoscopic (LRP) and Robotic (RARP)

The safety and efficacy of pure laparoscopic radical prostatectomy in RTRs has also been described (63, 64). Thomas and colleagues used a standard 6-port transperitoneal approach in three RTRs and successfully performed their dissection with harmonic scalpel and cold endoshears while using transrectal ultrasound to help guide the posterior prostatic dissection. They did not feel that the transplant ureteroneocystostomy affected their ability to complete the urethrovesical anastomosis or bladder neck transection. However, they did note some increased difficulty in dissecting and ligating the dorsal venous complex (64). Laparoscopic prostatectomy has also been successfully performed with an extraperitoneal approach (ERARP). In 2009, Robert and colleagues described ERARP in nine consecutive RTRs. They used two 10mm ports and three 5mm ports. When compared to the general population, ERARP in RTRs resulted in similar operative times, estimated blood loss, and oncologic outcomes. However, they did report a heightened risk of rectal injury (2 of 9 patients) (65).

In current practice, robotic assisted radical prostatectomy has quickly become the most frequently utilized surgical approach for prostatectomy. RARP has been successfully performed in a growing number of RTRs and seems to result in the least blood loss and shortest length of stay versus other surgical modalities. Important technical nuances have been described to optimize the safety of RARP in RTRs. Polcari et al. reported seven RARPs in RTRs between 2004-2010. They used a 4-arm robotic platform in all patients. Port adjustments included placement of the robotic 4th arm ipsilateral to the allograft and placement of two assistant ports contralateral to the allograft. Lymphadenectomy was performed only on the contralateral side. They recommend initiating bladder mobilization and subsequent development of the space of Retzius on the contralateral side of the allograft. Upon crossing the midline and working toward the allograft, a “fibrotic veil” is encountered that will contain the transplant kidney and ureter. The authors emphasize that this area should be avoided; the transplant ureter need not be visualized for safe completion of the procedure (66). Others have described a variety of port site modifications for RARP in RTRs. Jhaveri et al. advocate for use of an extended length bariatric assistant port ipsilateral to the allograft, which allows instruments to safely bypass the allograft by entering directly at the level of the prostate (67).

### Radiation Therapy

Radiation therapy has been used to treat CaP in RTRs, but is often avoided due to risks of allograft injury, ureteral injury, and urethral strictures. Mouzin et al. were the first to report the use of modern conformal prostate radiation therapy (XRT) in RTRs. Eight patients were treated with 70Gy in 2G fractions with 10mm margins and a 9-field arrangement. At a mean follow-up of 28 months, there were two biochemical recurrences (25%) and over half of the patients experienced short term minor morbidity including diarrhea, rectal irritation, cystitis, or hematuria. Although post-XRT creatinine clearance was unimpaired, significant ureteral obstruction occurred in two patients. The radiation exposure to the ureteroneocystostomy has been estimated to be between 20Gy and 40Gy, and is likely variable based on transplant ureteral orifice location (72). The safety of XRT for CaP in RTRs remains in question, as ureteral obstruction may enhance the risk of allograft dysfunction and may require future surgical procedures for revision. Some experts now view renal transplantation as a contraindication to prostate XRT given the risk of injury to the allograft and transplant ureter. Others maintain that radiation therapy is a safe, viable option in RTRs as doses delivered to the graft are typically below thresholds reported to induce complications and risk of ureteral injury can be minimized by ensuring the bladder is full at the time of irradiation (73).

### Brachytherapy

Compared to radical prostatectomy and EBRT, brachytherapy is thought to have less risk of damage to the allograft and transplant ureter. Although data is limited, these patients seem to have similar cancer control rates compared to the non-immunosuppressed population. Beydoun et al. reported successful brachytherapy in four RTRs with no PSA progression, morbidity, or altered allograft function at mean follow-up of 44 months (74). Similarly, Coombs et al. treated 17 immunosuppressed patients (including three RTRs) with brachytherapy and reported a long-term failure rate of 14.3%, which was not significantly different than age matched controls (15.8%) (75). Although data remains limited, current expert opinion supports the use of brachytherapy as a first line treatment option in RTRs with CaP who are over age 70 or are poor surgical candidates due to other comorbidities.

### Other treatment modalities

To date, there is a paucity of data regarding the use of other CaP treatment modalities (proton beam therapy, cryotherapy, high intensity focused ultrasound, hormonal therapy, stereotactic guided radiation therapy) in RTRs. One study by Al Ekish et al. reported successful use of prostate cryotherapy in 30 nonsurgical candidates, including two kidney/pancreas transplant recipients. Neither transplant patient experienced intraoperative complications, postoperative complications, or CaP recurrence after 18 months of follow-up (76). Overall, it remains unclear whether or not these modalities can be used in RTRs with comparable risk profiles and results compared to the non-transplant population. Finally, in patients with advanced prostate cancer in the setting of a kidney transplant, there are no guidelines for the administration or sequencing of the now multiple agents available, nor is there safety data for the agents in these clinical stage groupings. As such, these patients are treated based on the clinical judgment of the treating oncologist.

## CONCLUSIONS

CaP occurs with an increased incidence in RTRs and is diagnosed at an earlier age. As ESRD patients are being transplanted at older ages with improving allograft survival, the overall number of RTRs with CaP is increasing. The treatment team should pay special attention to PSA kinetics and immunosuppression in these patients. Older immunosuppressive agents (cyclosporine, AZA, tacrolimus) may increase risk of CaP, while newer agents (mTOR inhibitors) may decrease risk of CaP progression. Traditional prostate XRT should be used with caution in RTRs, as there is an increased risk of allo-graft and transplant ureteral injury. With special consideration and technical adjustments, surgical management of CaP in RTRs can be completed safely with similar morbidity and oncologic outcomes compared to the general population. In patients who are poor surgical candidates, prostate brachytherapy can also be performed safely with good oncologic outcomes. Further studies are needed to elucidate the risks and benefits of other CaP treatment strategies in the post-transplant population.
